# Structural Basis for Allostery in PLP-dependent Enzymes

**DOI:** 10.3389/fmolb.2022.884281

**Published:** 2022-04-25

**Authors:** Jenny U. Tran, Breann L. Brown

**Affiliations:** ^1^ Department of Biochemistry, Vanderbilt University School of Medicine, Nashville, TN, United States; ^2^ Center for Structural Biology, Vanderbilt University School of Medicine, Nashville, TN, United States

**Keywords:** pyridoxal 5-phosphate, allostery, enzyme structure, protein structural & functional analysis, coenzyme, protein-protein interaction

## Abstract

Pyridoxal 5′-phosphate (PLP)-dependent enzymes are found ubiquitously in nature and are involved in a variety of biological pathways, from natural product synthesis to amino acid and glucose metabolism. The first structure of a PLP-dependent enzyme was reported over 40 years ago, and since that time, there is a steady wealth of structural and functional information revealed for a wide array of these enzymes. A functional mechanism that is gaining more appreciation due to its relevance in drug design is that of protein allostery, where binding of a protein or ligand at a distal site influences the structure, organization, and function at the active site. Here, we present a review of current structure-based mechanisms of allostery for select members of each PLP-dependent enzyme family. Knowledge of these mechanisms may have a larger potential for identifying key similarities and differences among enzyme families that can eventually be exploited for therapeutic development.

## 1 Introduction

Pyridoxal 5′-phosphate (PLP), the active form of vitamin B6, is a coenzyme that is required for the activity of many proteins from bacteria to humans. These enzymes carry out a number of processes, including amino acid metabolism and biosynthesis of antibiotic compounds (Schneider et al., 2000). PLP-dependent enzymes typically bind the cofactor covalently via a conserved catalytic lysine residue, forming an internal aldimine. After binding the amino acid substrate, the internal aldimine is exchanged for an external aldimine Schiff base with the amino group of the substrate and the PLP aldehyde, regenerating the free lysine (Schneider et al., 2000). The reaction then proceeds through a quinonoid intermediate with the PLP cofactor providing an electron sink to stabilize the transient reaction intermediates (Eliot and Kirsch 2004). After these initial steps, there is a diverse number of reactions PLP-dependent enzymes mediate, including decarboxylation, transamination, and racemization (Liang et al., 2019). In fact, PLP-dependent enzymes are known to mediate more than 140 distinct activities (Percudani and Peracchi 2003).

**FIGURE 1:**
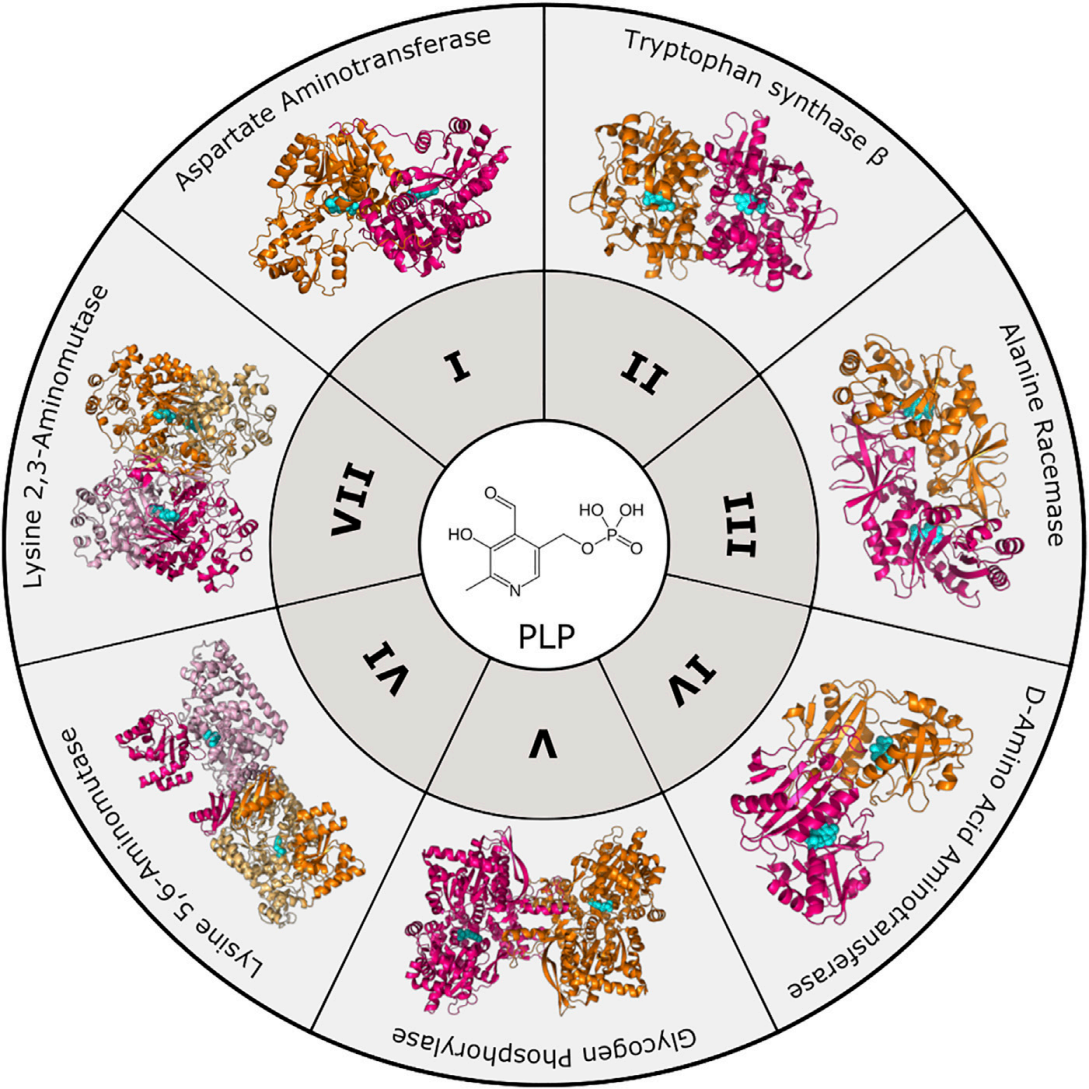
PLP-dependent enzyme families. To date, PLP-dependent enzymes are categorized into seven different families based on structural homology to an archetypal enzyme. These include Aspartate Aminotransferase (Fold Type I, PDB 8AAT), Tryptophan Synthase β-subunit (Fold Type II, PDB 1BKS), Alanine Racemase (Fold Type III, PDB 1SFT), D-Amino Acid Aminotransferase (Fold Type IV, PDB 1DAA), Glycogen Phosphorylase (Fold Type V, PDB 1GPB), Lysine 5,6-Aminomutase (Fold Type VI, PDB 1XRS), and Lysine 2,3-Aminomutase (Fold Type VII, PDB 2A5H). Protomers are colored orange and magenta with the PLP cofactor shown in cyan.

To date, there are seven PLP-dependent protein families that are classified based on their 3-dimensional folds (Figure 1). Although proteins within each family have little overall sequence homology, they exhibit characteristic structures. Notably, a particular fold does not necessarily dictate a particular reaction, as each fold type can mediate multiple types of reactions [for review see (Eliot and Kirsch 2004)]. Fold Type I is the largest family typified by the enzyme aspartate aminotransferase. These enzymes are homodimers with each protomer containing a large and small subdomain; however, the PLP-binding sites are comprised of residues from both subunits. Although structurally similar to Type I, Fold Type II, or the tryptophan synthase β family, typically contains enzymes that catalyze β-elimination, β-replacement, and racemization reactions. They differ from Fold Type I in that the active sites are usually made up of residues from only one protomer, and there may be additional regulatory domains present. Fold Type III (alanine racemase) enzymes, characterized by an α/β-barrel attached to a β-strand domain, also function as dimers and include several amino-acid decarboxylases. Fold Type IV enzymes include D-amino acid aminotransferase and a few other enzymes that also function as homodimers, but the PLP-binding mode differs from Folds I and II. The Fold Type V group includes glycogen phosphorylase and is atypical from other families in that this clade uses the PLP phosphate group for catalysis (Eliot and Kirsch 2004). More recently, two new PLP-dependent families were identified with structural divergence from the other families, and they are denoted Fold Type VI (lysine 5,6-aminomutase) and Fold Type VII (lysine 2,3-aminomutase) (Percudani and Peracchi 2009).

In addition to structural diversity, PLP-dependent enzymes also display a diverse repertoire of allosteric mechanisms. Protein allostery is key for the modulation of enzyme activity given a particular cellular context or binding partner. Protein allostery, a term first coined 60 years ago (Monod and Jacob 1961), is defined as the ability for ligand or protein binding at one site to affect the binding or activity of another distal site (Monod et al., 1965). A seminal example of allostery is the Bohr effect, where factors affecting blood pH cause a change in the binding affinity of hemoglobin for oxygen. Since these early studies, allosteric control has been identified for many proteins mediating nearly all functions in the cell. Along with the expansion of our understanding of allostery, the methods to determine and study allostery have also grown. Regardless of the mechanism, allostery presents a powerful tool for proteins to regulate their own functions and can even be harnessed for drug design (Nussinov and Tsai 2013). This review will focus on the various allosteric mechanisms and their structural bases invoked by members of each PLP-dependent enzyme family. Although we focus on a set of key proteins, PLP-dependent enzymes exhibit a broad range of allosteric mechanisms in addition to these specific examples (Supplementary Table S1). Future work in therapeutic development will benefit from a thorough understanding of these burgeoning principles.

## 2 Diverse PLP-dependent Proteins and Their Allosteric Mechanisms

### 2.1 Fold Type I: 5-Aminolevulinic Acid Synthase (ALAS)

**FIGURE 2:**
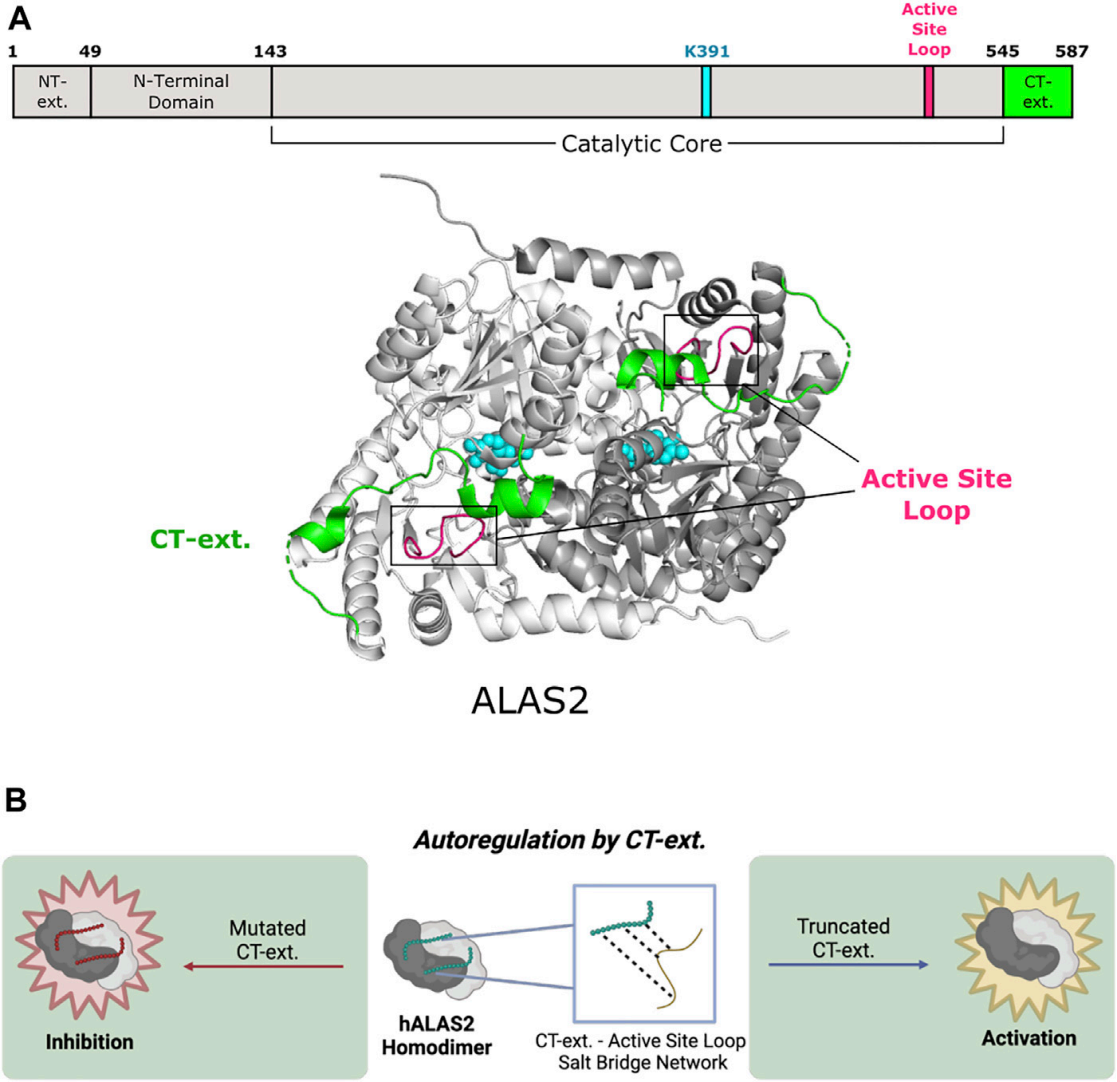
Human 5-aminolevulinic acid synthase 2 of Fold Type I undergoes autoregulation via its C-terminal extension. **(A)** Human ALAS2 (PDB 6HRH) has a C-terminal extension (CT-ext.; aa 545–587; green) that interacts with the active site loop (aa 500–517; pink) from the catalytic core to block access to the active site. ALAS2 protomers are shown in white and gray. PLP and the active site lysine (K391) are shown in cyan. **(B)** The CT-ext. of ALAS2 exhibits an autoregulatory mechanism by occluding the active site. Truncation of the CT-ext. disrupts the salt bridge network between the CT-ext. and active site loop, leading to gain of function. Mutations of the CT-ext. may also cause loss of function by changing the active site conformation (figure created with BioRender.com).

PLP-dependent enzymes belonging to Fold Type I exhibit a conserved structure typical of many aminotransferases, decarboxylases, and enzymes that catalyze α-, β- or γ-eliminations (Percudani and Peracchi 2003). 5-aminolevulinic acid synthase (ALAS) is the first and rate-limiting enzyme for heme biosynthesis in α-proteobacteria and the mitochondria of non-plant eukaryotes. ALAS catalyzes the condensation of glycine and succinyl-CoA to yield aminolevulinic acid (Gibson et al., 1958; Kikuchi et al., 1958; Laver et al., 1958). ALAS is a member of the α-oxoamine family of Fold Type I PLP-dependent enzymes (Schneider et al., 2000; Eliot and Kirsch 2004) and exists as a homodimer with the two PLP cofactor binding pockets buried at the subunit interface (Figure 2A) (Astner et al., 2005). Currently, there are several published structures of ALAS enzymes from multiple organisms either bound covalently (internal aldimine) or non-covalently to PLP. The crystal structures of ALAS from *Rhodobacter capsulatus* were captured with both the internal aldimine (PDB 2BWN) and the glycine-bound external aldimine (PDB 2BWP) (Astner et al., 2005). Subsequently, ALAS structures from *Saccharomyces cerevisiae* and the erythroid-specific *Homo sapiens* isoforms were also crystallized in the presence of PLP, either bound covalently or non-covalently (Brown et al., 2018; Bailey et al., 2020). Importantly, these structures revealed the position and conformation of the eukaryote-specific ALAS C-terminal extension—a region absent from bacterial ALAS enzymes.

#### 2.1.1 C-Terminal Extension

The eukaryotic ALAS C-terminal extension allosterically communicates to the enzyme active site by controlling the position of the ALAS active site loop (Fratz et al., 2015; Bailey et al., 2020). The conformation and dynamics of this loop are reported to control the overall rate of ALA product release, which is the rate-limiting step of the ALAS reaction (Hunter and Ferreira 1999; Astner et al., 2005; Hunter et al., 2007; Lendrihas et al., 2010). In yeast ALAS, the extreme portion of the C-terminus makes *trans* interactions with the neighboring subunit via an interaction with a conserved arginine residue located in the ALAS catalytic glycine-rich motif. Importantly, mutation of this arginine in yeast or eukaryotic ALAS leads to a decrease in enzyme activity (Gong and Ferreira 1995; Katsurada et al., 2016; Brown et al., 2018). Thus, for yeast ALAS, the C-terminus has two points of allosteric control—first by regulating the position of the active site loop and second by interacting with the ALAS glycine-rich loop adjacent to the enzyme’s active site (Brown et al., 2018). The mammalian C-terminal extension also allosterically regulates enzyme activity of ALAS2, the erythroid-specific isoform (Figure 2B). Notably, deletion or modification of this region in humans underlies toxic hyperactivity leading to porphyrin accumulation and the disease X-linked protoporphyria (XLP) (Whatley et al., 2008; Bishop et al., 2013; Ducamp et al., 2013). The recent crystal structure of human ALAS2 identified key interactions between the C-terminal extension and other regulatory regions of the enzyme (Bailey et al., 2020). A short helix in this extension (helix α15, Ser568-Phe575) forms a lid over the active site but does not directly contact the non-covalently bound PLP cofactor. Nonetheless, *in vitro* biochemical experiments showed that disruption of the human C-terminal extension alters the PLP microenvironment and changes the tautomeric equilibrium of the cofactor (Fratz et al., 2015). Additionally, it was determined that the orientation of the internal aldimine in the XLP variants is different from wild-type ALAS2 in the presence of bound succinyl-CoA substrate. The ALAS2 crystal structure reveals a direct interaction between an arginine in the active site loop and the C-terminal extension, leading to the hypothesis that the conformation and flexibility of the active site loop are coupled to changes in the C-terminus, thus controlling overall ALAS2 activity (Bailey et al., 2020). Finally, the ALAS2 C-terminal extension may auto regulate enzyme activity by acting as a signal for degradation (Kadirvel et al., 2012). Thus, the eukaryote-specific ALAS C-terminal extension acts as a homo-allosteric regulator of enzyme activity at multiple nodes.

### 2.2 Fold Type II: Cystathionine β-Synthase (CBS)

**FIGURE 3:**
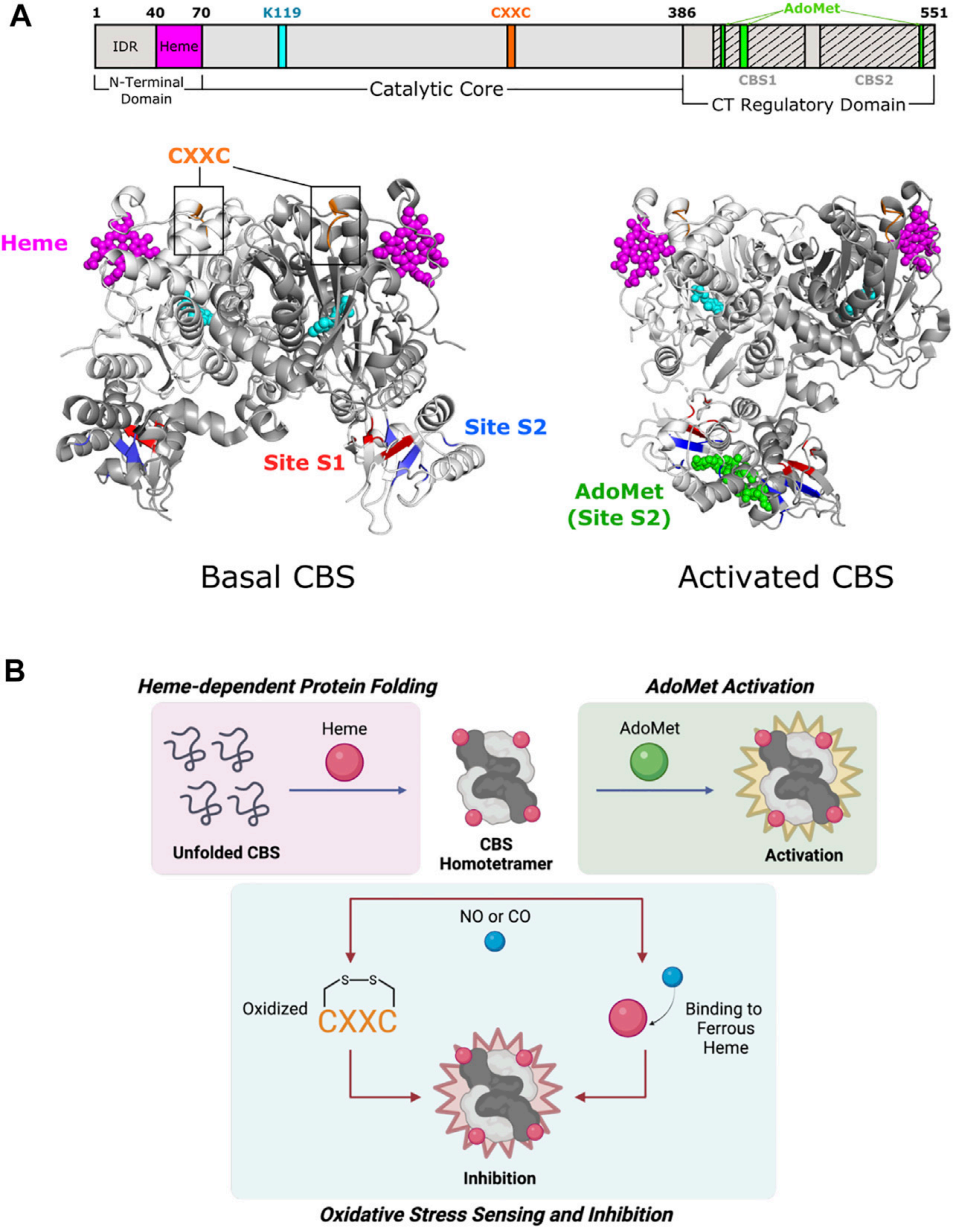
Cystathionine β-synthase of Fold Type II involves each of its domains in allostery. **(A)** Human CBS contains three domains that are all implicated in allostery. Heme binding occurs in a shallow pocket (aa 40–70; magenta) at the N-terminal domain. The catalytic core contains a CXXC oxidoreductase motif (aa 272–275; orange). The C-terminal regulatory domain has a Bateman module consisting of two tandem CBS motifs, CBS1 (aa 412–471) and CBS2 (aa 477–551), which fold to form Site S1 (M458, V459, Y484, F487, F508, and A509; red) and Site S2 (P422, L423, F443, A446, P447, V448, V533, and V534; blue) for AdoMet binding. Basal CBS (PDB 4L0D) has little activity due to the regulatory domain of one subunit blocking access to the active site of a neighboring subunit. Activated CBS (PDB 4PCU) binds AdoMet (green) in Site S2, which moves the regulatory domain away from the active sites to allow access. CBS protomers are shown in white and gray. PLP and the active site lysine (K119) are shown in cyan. **(B)** Formation of the CBS homotetramer is mediated by heme binding, in which the heme porphyrin scaffold facilitates protein folding. AdoMet binds and activates the CBS homotetramer. CBS inhibition takes place via oxidation of the CXXC motif and/or gaseous signaling molecule (i.e., NO, CO) binding to ferrous heme.

Fold Type II (also known as the tryptophan synthase β family) encompasses numerous allosteric enzymes (Liang et al., 2019), including the tryptophan synthase α_2_β_2_ complex (Hyde et al., 1988), threonine deaminase (Eisenstein 1995), threonine synthase (Curien et al., 1998), and O-acetylserine sulfhydrylase (Burkhard et al., 2000). Although enzymes in this fold have active sites composed of residues from one subunit, they are active in various oligomeric states (usually dimers or tetramers) that also accommodate allosteric regulation (Gallagher et al., 1998; Garrido-Franco et al., 2002; Fatmi and Chang 2010). For example, fungal threonine synthase functions as a monomer and is not subject to allosteric regulation, whereas plant threonine synthase is found as a homodimer and is activated by S-adenosyl-L-methionine (AdoMet) (Garrido-Franco et al., 2002). Cystathionine β-synthase (CBS) is involved in the initial step of sulfur-containing amino acid biosynthesis (Gerritsen and Waisman 1964) where it catalyzes the condensation of serine and potentially toxic homocysteine to yield cystathionine. It assembles as a tetramer (a dimer of dimers), with each subunit consisting of a catalytic N-terminal domain that binds PLP and heme and a regulatory C-terminal domain (Figure 3A) (Taoka et al., 2002). In addition to PLP, CBS uses heme as a cofactor and is further activated by AdoMet. CBS distinguishes itself from other family members in how both the N-terminal and C-terminal domains participate in allostery (Meier et al., 2001).

#### 2.2.1 C-Terminal Regulatory Domain

Alignment of Fold Type II enzymes show a highly conserved catalytic core and minimally conserved N- and C-terminal extensions (Miles and Kraus 2004). A key feature of allosteric enzymes in this fold is the C-terminal regulatory domain, which is usually involved in effector binding (Gallagher et al., 1998; Garrido-Franco et al., 2002). In CBS, truncation of this domain yields the “active core” that is not activated by AdoMet, has twice the enzymatic activity of full-length CBS, and forms dimers instead of tetramers (Meier et al., 2001). Available CBS crystal structures only represent mutant CBS dimers, whereas native wildtype CBS exists as tetramers (Ereno-Orbea et al., 2013).

The C-terminal regulatory domain of human CBS has two tandem β-α-β-β-α secondary structure motifs known as “CBS domains” that can be found in other proteins (Bateman 1997; Ignoul and Eggermont 2005). These motifs interact to form intramolecular structures known as Bateman modules or CBS pairs. PLP is deeply buried in a cleft between the N-terminal and C-terminal domains of a subunit (Meier et al., 2001). The CBS1 (amino acids 415–468) and CBS2 (aa 511–531) domains from one subunit associate to block the narrow active site channel of a neighboring subunit and form AdoMet binding clefts called Site S1 and Site S2 (Figure 3A). Site S1 is blocked by hydrophobic residues, leaving Site S2 as the effective AdoMet binding site ∼10 Å from the active site. Site S2 is therefore known as an autoinhibitory region, where AdoMet binding displaces the regulatory domain of one subunit from the catalytic cavity of another subunit. Rotation of Site S2 occurs and weakens interactions with the loops of the catalytic cavity. The active site thus becomes less sterically hindered and is kinetically stabilized (Ereno-Orbea et al., 2013). Because of its effect on active site accessibility, AdoMet is known as a V-type allosteric activator (which increases V_max_) and can bind to each subunit to cause a 2- to 3- fold increase in activity (Taoka et al., 1999a).

#### 2.2.2 Heme Binding Domain

CBS is one of few PLP-dependent enzymes that binds heme for regulatory purposes. Heme is not essential for catalysis in yeast and parasitic CBS as they are active but do not bind heme (Jhee et al., 2000; Nozaki et al., 2001). However, alterations in heme binding modulate human CBS activity and stability (Badawy 2021). The N-terminal domain of CBS comprises two distinct regions. The first few residues contain an intrinsically disordered region (IDR) that may play a part in heme binding. The IDR has a canonical cysteine-proline (CP) motif that is found in other heme-binding proteins such as human ALAS enzymes, but the function of this site in CBS is unknown (Kumar et al., 2018). The remaining residues in the N-terminal domain fold into a shallow, hydrophobic pocket known to non-covalently bind heme ∼20 Å from the active site (Figure 3A) (Meier et al., 2001).

The mechanism of heme regulation is not fully understood, but the components of heme may play different roles. The porphyrin moiety of heme acts as a scaffold to facilitate protein folding and maintain stability (Majtan et al., 2008). Alternatively, the heme iron may be involved in oxido-reducing reactions that allow for proper enzyme function (Figure 3B) (Taoka et al., 1998). Redox sensitivity is an important property in determining CBS activity since the metabolism of homocysteine is directly related to cellular redox homeostasis (Weiss et al., 2002; Prudova et al., 2006). The heme iron can exist in two redox states, Fe(III)-CBS (ferric heme) and the reduced Fe(II)-CBS (ferrous heme) (Carballal et al., 2013). Ferrous heme binds available gaseous signaling molecules (e.g., NO or CO) with relatively high affinity (Taoka et al., 1999b; Vicente et al., 2014), which inhibits CBS activity by up to 2-fold compared to ferric CBS (Taoka et al., 1998). A possible pathway for allosteric regulation by heme is through the formation of salt bridges, a mechanism that has been shown to be important in conformational changes of other heme proteins. In CBS, Arg266 is located at the distal end of a helix that makes hydrogen bonds with the phosphate moiety of PLP. This same arginine forms a salt bridge with Cys52, a heme-binding residue, which allows for conformational changes to propagate to the active site (Taoka et al., 2002).

Certain data questions the existence of the ferrous form of CBS *in vivo* because of the low redox potential of heme in wild-type CBS (Singh et al., 2009). However, it was reported that methionine synthase reductase can indeed reduce the CBS heme in an NADPH-dependent manner, supporting the biological relevance of ferrous CBS (Kabil et al., 2011). Another potential site for redox sensing is an oxidoreductase motif, CXXC, located ∼20 Å away from the active site. Mutagenesis of either cysteine within the motif affects CBS activity, but overall structural changes have not yet been elucidated due to the lack of a structure for full-length, oxidized CBS (Niu et al., 2018).

### 2.3 Fold Type III: Ornithine Decarboxylase (ODC)

**FIGURE 4:**
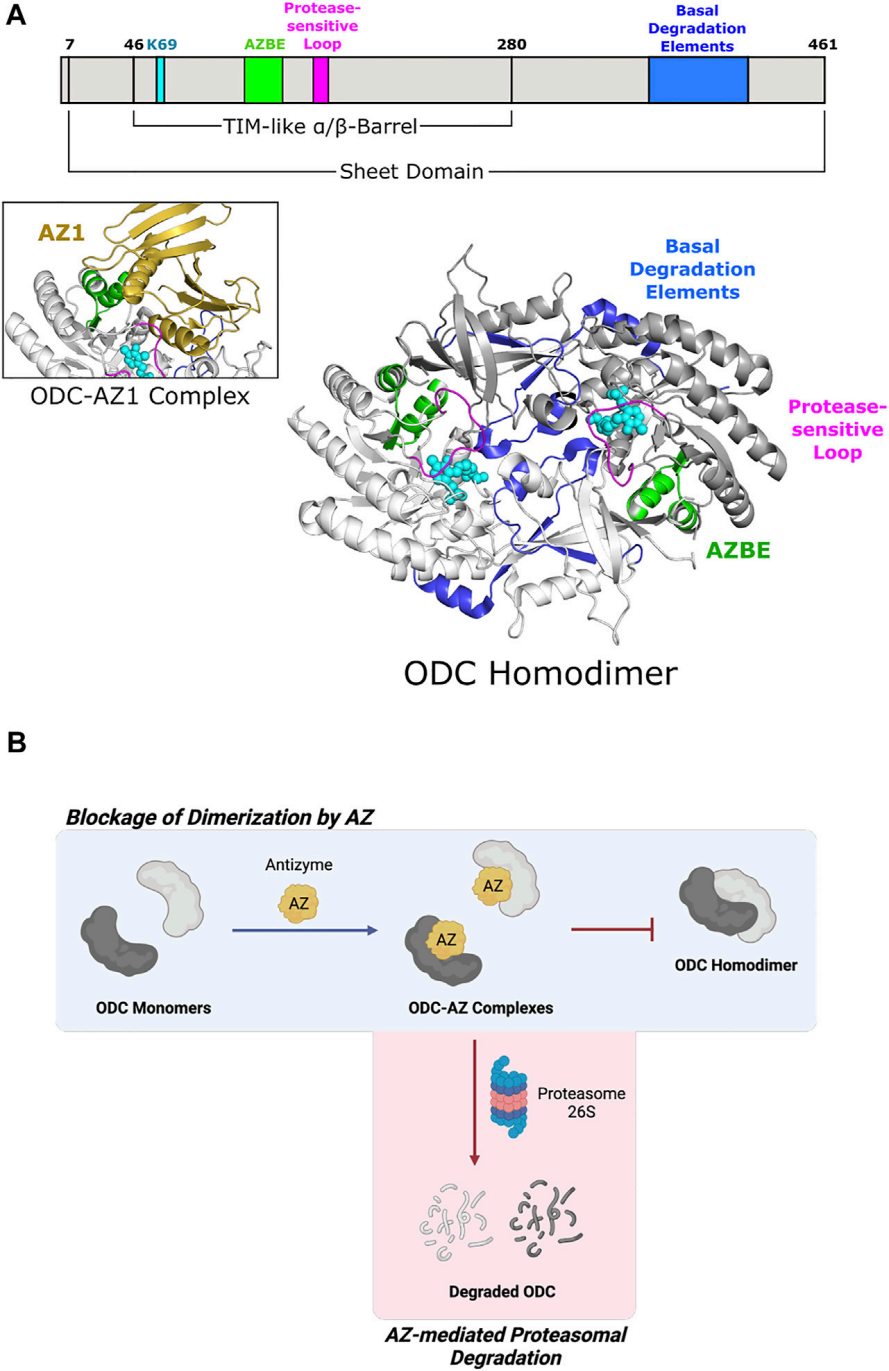
Eukaryotic ornithine decarboxylase of Fold Type III is targeted for proteasomal degradation by interaction with Antizyme 1. **(A)** Human ODC (PDB 1D7K) binds AZ1 at the antizyme binding element (AZBE; aa 117–140; green) to form an ODC-AZ1 heterodimer (AZ1 in yellow; PDB 4ZGY). Two basal degradation elements (aa 376–427; blue) are subsequently exposed to allow ubiquitin-independent, proteasomal degradation of ODC. A protease-sensitive loop (aa 158–168; magenta) positions active site residues for catalysis. ODC protomers are shown in white and gray. PLP and the active site lysine (K69) are shown in cyan. **(B)** ODC dimerization is blocked by Antizyme 1 binding, leading to ODC-AZ1 heterodimer formation. This inactive form of ODC is then targeted by the 26S proteasome for degradation.

Fold Type III, or the alanine racemase family, is characterized by a mixed α/β barrel structure found in certain amino-acid decarboxylases (Percudani and Peracchi 2003). L-Ornithine decarboxylase (ODC), a member of the Group IV family of decarboxylases, catalyzes the first and rate-limiting step of polyamine biosynthesis, which is the formation of putrescine from ornithine (Sandmeier et al., 1994). Putrescine is ultimately converted into the polyamines spermidine and spermine (Gale 1940; Tabor and Tabor 1976; Cohen 1998; Pegg 2006). There are structures of ODC enzymes from multiple organisms, including bacteria and mammals. Each ODC subunit contains an N-terminal PLP-binding domain with a TIM-like α/β-barrel fold and a C-terminal β-sheet domain (Figure 4A) (Kern et al., 1999; Almrud et al., 2000). The active sites, which accommodate PLP and L-ornithine, are formed at the dimer interface between the N-terminal domain of one subunit and the C-terminal domain of the partner subunit (Almrud et al., 2000; Jackson et al., 2004). Wild-type ODC dimers are weakly-interacting and exist in equilibrium with the monomeric form (Pegg 2006).

#### 2.3.1 Antizyme Binding

Antizyme is a non-competitive protein inhibitor of ODC that is produced in response to an increase in cellular polyamine levels (Heller et al., 1976). Antizyme binds to the free ODC monomer, forming an inactive heterodimer (Fujita et al., 1984) that sterically blocks the ODC homodimerization interface (Figure 4B) (Wu et al., 2015). Antizyme also abrogates ODC function by increasing the interaction of ODC with the proteasome in a ubiquitin-independent manner (Murakami et al., 1992). The crystal structure of human ODC in complex with a portion of antizyme isoform 1 indicates a conformational rearrangement in ODC which may reveal a structural feature that is then recognized by the proteasome (Wu et al., 2015) (Figure 4A). The C-terminal extension of ODC, which is absent from *Trypanosoma brucei* ODC (Persson et al., 2003), represents another point of allosteric control via serving as a signal for degradation by the 26S proteasome. Experiments using murine ODC showed deletion of the C-terminal 37 amino acids prevents proteasomal degradation (Ghoda et al., 1989). Additional work showed this peptide serves as a protein degron as appending it to *T. brucei* ODC leads to degradation (Zhang et al., 2003). Although not required for proteasome binding, the ODC C-terminus is necessary for degradation as a truncated version was stable even in the presence of antizyme (Wu et al., 2015). Unfortunately, there is no structural information pertaining to this region since it remains disordered in known mammalian ODC crystal structures (Almrud et al., 2000; Wu et al., 2015). How this region is recognized by the proteasome remains an outstanding question in the field.

#### 2.3.2 Allosteric Inhibitors

In addition to homo-allostery, much work is focused on developing direct and allosteric ODC inhibitors since it promotes cell transformation and is overexpressed in many cancers (Pegg 1988; Auvinen et al., 1992; O'Brien et al., 1997). Additionally, *T. brucei* ODC is a validated drug target to treat African Sleeping Sickness (trypanosomiasis) (Barrett et al., 2007; Smithson et al., 2010). Difluoromethylornithine (DFMO) is a structural analog of L-ornithine that works as an irreversible suicide ODC inhibitor by binding at the active site and forming a covalent adduct between the PLP cofactor and the conserved Cys360 (Metcalf et al., 1978; Poulin et al., 1992; Grishin et al., 1999); this same residue is also affected by S-nitrosylation via nitric oxide treatment (Bauer et al., 2001). Unfortunately, DFMO exhibits a low affinity for ODC and high doses of DFMO can result in permanent hearing loss (Lao et al., 2004), so ongoing work is targeted toward finding inhibitors with lower toxicity. To this end, multiple groups have located allosteric inhibitory sites on both *T. brucei* and human ODC. For example, Geneticin is a weak, non-competitive inhibitor that binds at the interface between the ODC N- and C-terminal domains, inducing an order-to-disorder transition in a key catalytic loop located at the dimer interface (Jackson et al., 2003). Herbacetin is a natural product that was shown through computational modeling and *in vitro* studies to bind at an allosteric site on ODC (comprised of residues Asp44, Asp243, and Glu384) to inhibit ODC activity (Kim et al., 2016). Another natural product, allicin, reversibly S-thioallylates accessible ODC cysteines, causing reduced polyamine levels and cell proliferation (Schultz et al., 2020) It is unclear whether the allicin-induced deactivation of ODC is due to thioallylation of the conserved Cys360, disruption of ODC dimerization, or some other unknown mechanism. More recent work is directed toward characterizing multipurpose inhibitors that might inhibit ODC’s activity, target the ODC-Antizyme1 interaction, and enhance non-functional ODC dimerization (Chai et al., 2020)

### 2.4 Fold Type IV: Branched-chain L-amino Acid Aminotransferase (BCAT)

Unlike other fold types, the D-amino acid aminotransferase family is not yet reported to contain enzymes that display clear allostery. Fold Type IV consists of four broad categories: (*S*)-selective branched-chain L-amino acid aminotransferases (BCATs) (Taylor and Jenkins 1966; Grishin et al., 1995), (*R*)-selective D-amino acid aminotransferases (DAATs) (Peisach et al., 1998), (*R*)-amine:pyruvate transaminases (R-ATAs) (Iwasaki et al., 2012), and 4-amino-4-deoxychroismate lyases (ADCLs) (Nakai et al., 2000). Out of these four protein types, the first three are transaminases. Although Fold Type I also contains transaminases that use similar enzymatic mechanisms, those of Fold Type IV have strict (*R*)- or (*S*)-stereospecificity and orient PLP differently in the active site (Okada et al., 1997; Bezsudnova et al., 2020).

**FIGURE 5:**
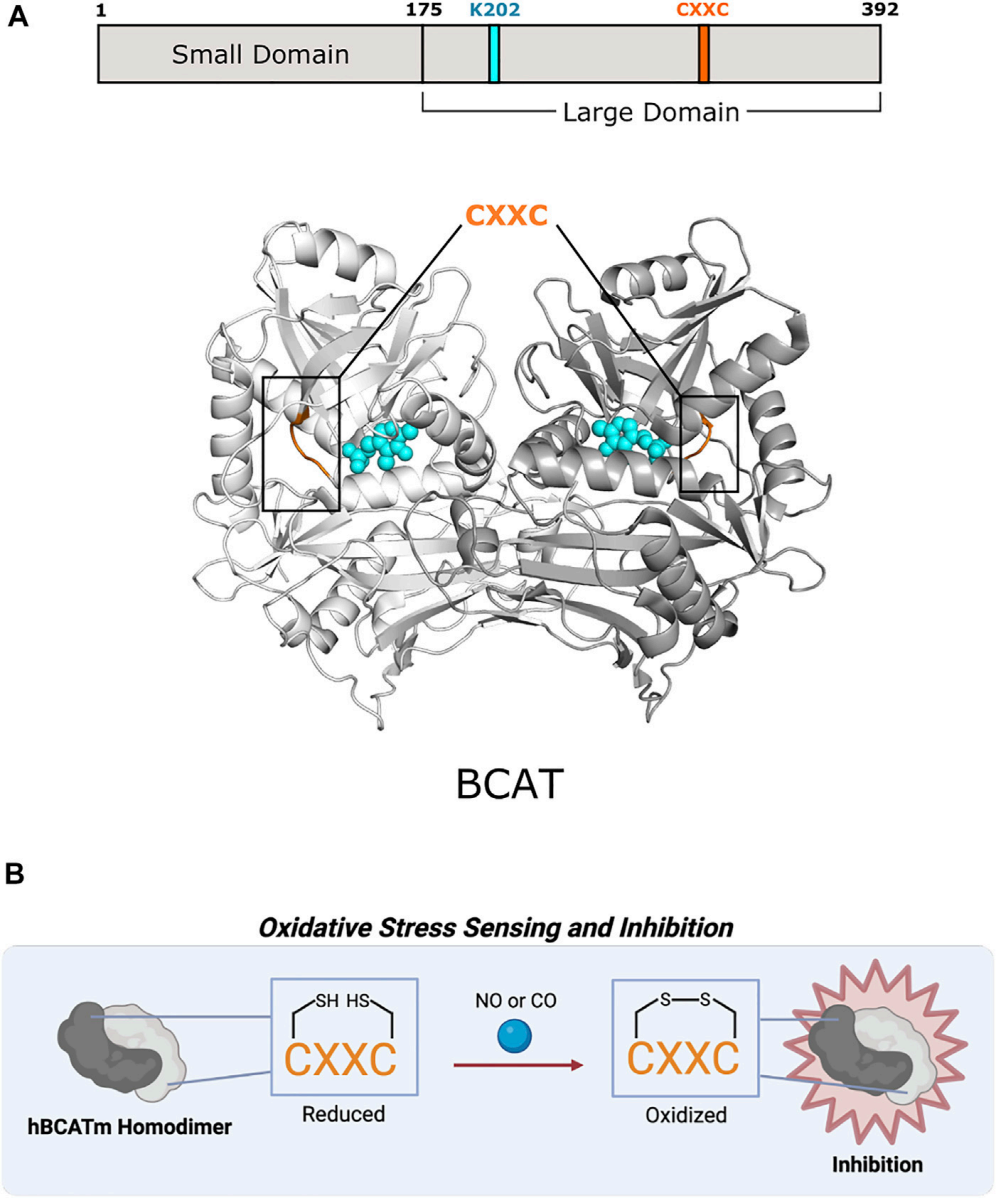
Human mitochondrial branched-chain amino acid aminotransferase of Fold Type IV uses a CXXC motif for preventing overoxidation. **(A)** Human mitochondrial BCAT (PDB 1EKF) has a CXXC motif (aa 315–318; orange) in which one cysteine (C315) acts as an oxidative sensor and the other (C318) is a “resolving cysteine” to prevent overoxidation of hBCATm. BCAT protomers are shown in white and gray. PLP and the active site lysine (K202) are shown in cyan. **(B)** BCAT is inhibited via oxidation of the CXXC motif.

Generally, most Fold Type IV enzymes form homodimers for catalysis (Sugio et al., 1995; Bezsudnova et al., 2020), but some BCATs and R-ATAs form tetramers or hexamers (Inoue et al., 1988; Iwasaki et al., 2012; Isupov et al., 2019). It is not known if these different oligomeric states are important for function. In an active dimer, one subunit comprises two domains connected by an interdomain loop (Figure 5A). The small N-terminal domain has an α/β structure. The large C-terminal domain has a pseudo β-barrel structure. The active site exists at the bottom of the subunit interface cleft formed by residues from both domains of one subunit and the small domain of a neighboring subunit (Okada et al., 2001). Although the active site is geometrically similar within proteins of this family, strict stereospecificity is based on different amino acid compositions (Bezsudnova et al., 2020). Another important note is that most enzymes belonging to this family are bacterial, archaeal, or plant proteins, and only BCATs have mammalian homologs (Ichihara and Koyama 1966; Taylor and Jenkins 1966). There has been a focus on industrial, antibiotic, and herbicidal applications because of the unique stereospecificity that can be exploited with Fold Type IV enzymes (Nakai et al., 2000; Pavkov-Keller et al., 2016). Therefore, much industrial research has focused on using the differences in active sites to obtain stereoselective products.

Excluding antibiotic development, therapeutic efforts have only extended toward BCATs. In humans, branched-chain amino acids are nutrient signals, so BCATs are important facets in cancer and metabolic diseases (Lynch and Adams 2014; Hattori et al., 2017). BCAT inhibitor design has concentrated on obstructing reaction mechanisms, leading mainly toward irreversible, competitive inhibitors (Deng et al., 2015). Studies have shown that mammalian BCATs have an oxidoreductase CXXC motif that could be further investigated as a potential allosteric site as found in CBS in Fold Type II (Figure 5B). Humans have two BCAT isozymes, a mitochondrial form (hBCATm) and a cytosolic form (hBCATc). In hBCATm, the two cysteines of ^315^CXXC^318^ participate in thiol-thiolate interaction and are responsible for redox sensitivity. Cys315 acts as a sensor for redox regulation and helps in substrate orientation (Yennawar et al., 2006). Cys318 is a “resolving cysteine” that forms a reversible disulfide bond to prevent overoxidation or irreversible oxidation to sulfinic or sulfonic acid (Conway et al., 2004). Oxidation of the CXXC motif located ∼10 Å from the active site disrupts the hydrogen-bonding network necessary for PLP coordination and substrate channeling, thus inhibiting hBCATm (Yennawar et al., 2006). This motif also allows hBCAT to play a potential role as a redox chaperone in protein misfolding of neurodegenerative diseases like Alzheimer’s disease (El Hindy et al., 2014). There are currently no therapeutics that target the CXXC motif of mammalian BCATs, but further exploration into its function and role may provide new avenues for drug discovery.

### 2.5 Fold Type V: Glycogen Phosphorylase (GP)

This fold type contains only glycogen phosphorylase (GP), which is the first PLP-dependent enzyme to be structurally determined (Weber et al., 1978). Fold Type V enzymes use the phosphate moiety of PLP for proton transfer, whereas Fold Types I through IV use PLP as an electrophilic sink. As a result, the active site is completely divergent from other folds and binds PLP in a unique way (Schneider et al., 2000). GP is a well-known allosteric enzyme that, with the help of inorganic phosphate, catalyzes the phosphorolytic cleavage of α-1,4-glycosidic bonds to liberate the terminal glucose (glucose 1-phosphate; G1P) of a glycogen molecule in glycogenolysis (Hestrin 1949; Sprang et al., 1991). In mammals, glycogen is the main carbohydrate source and is found throughout the body, however, its function is tissue-dependent. GP has three isozymes, liver GP (lGP), muscle GP (mGP), and brain GP (bGP) (David and Crerar 1986; Newgard et al., 1988). Although they are encoded by separate genes, they are highly similar in sequence and differ mostly in expression and regulation.

#### 2.5.1 Ser14 Phosphorylation

**FIGURE 6:**
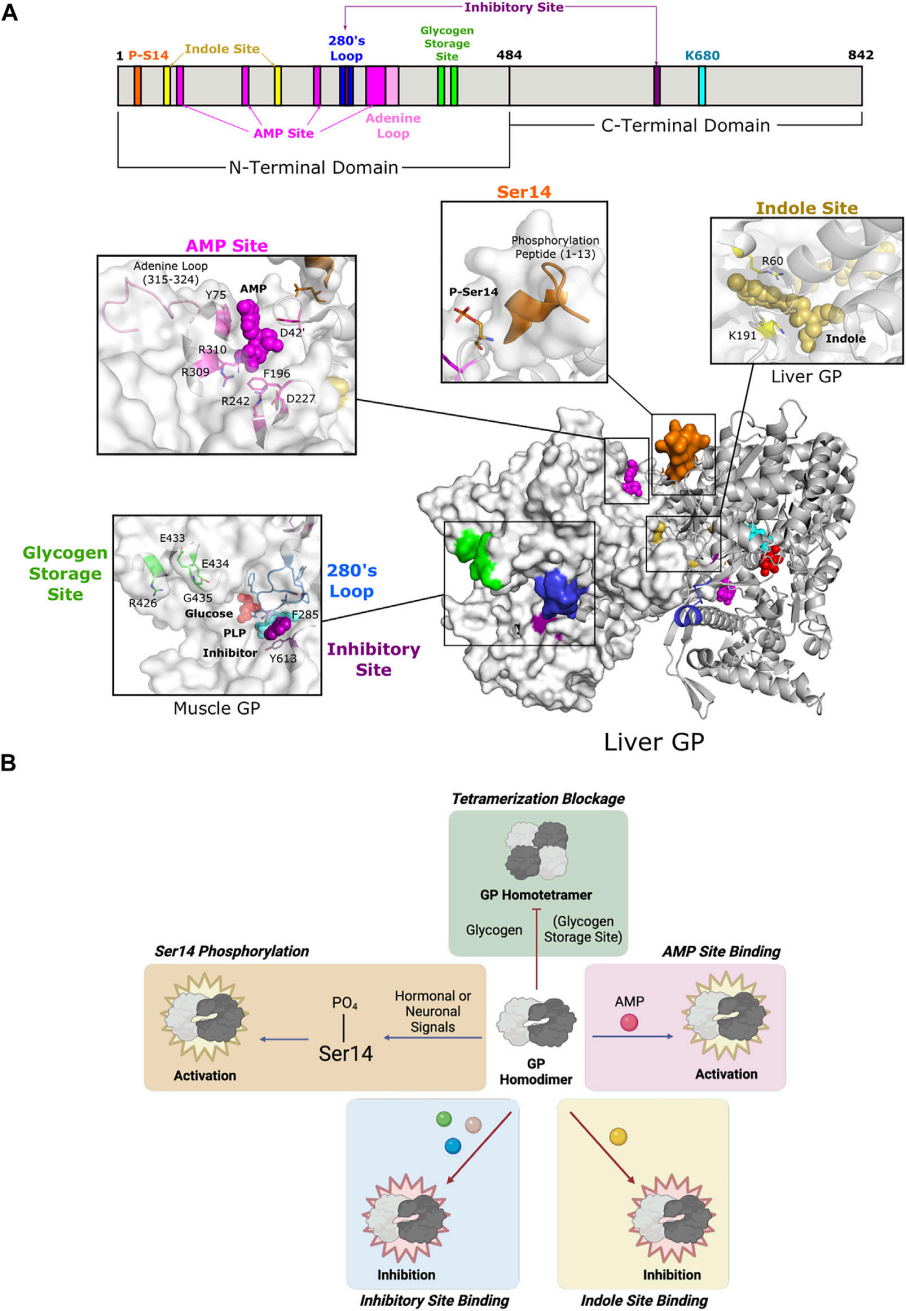
Glycogen phosphorylase of Fold Type V utilizes diverse modes of allostery. **(A)** One protomer of human liver GP homodimer (PDB 1FA9) is shown as a surface representation (white) and the second as a cartoon representation (gray). GP is phosphorylated at Ser14 (orange), which causes the phosphorylation peptide (aa 1–13; orange) to stabilize the active conformation. Ligand binding may occur at three sites: the AMP site, the indole site, and the inhibitory site. AMP binding at the AMP site (D42 of one subunit; Y75, F195, D227, R242, R309, and R310 of the other subunit; dark pink) is coordinated by the adenine loop (aa 315–324; light pink). The indole site (human liver GP, PDB 1XOI; R60, and K191; yellow) is located at the dimer interface. The inhibitory site (human muscle GP, PDB 1Z8D; F285, Y613; purple) is a shallow pocket at the entrance of the active site. The inhibitory site is also part of the 280s loop (aa 280–289; blue), which forms a gate for the active site. The glycogen storage site (R426, E433, E434, and G435; green) is part of the tetrameric interface (not shown). PLP and the active site lysine (K202) are shown in cyan. **(B)** GP is active as a homodimer and can be further activated via phosphorylation at Ser14 and/or AMP binding. Phosphorylation and AMP binding triggers GP tetramerization, which inactivates GP, but tetramerization can be blocked by glycogen binding. GP can also be inhibited by ligand binding at the indole and inhibitory sites.

One of the main allosteric mechanisms of glycogen phosphorylase is phosphorylation at Ser14 located ∼45 Å from the active site (Figure 6B) (Krebs and Fischer 1956; Sprang et al., 1991). Unphosphorylated GP is dependent on adenosine monophosphate (AMP) for activity and inhibited by glucose-6-phosphate (G6P) and adenosine triphosphate (ATP). However, phosphorylation causes the 22 amino-terminal residues of GP to become ordered, which induces the rotation of subunits within the functional dimer to activate the enzyme and further enhance AMP activation (Barford and Johnson 1989; Newgard et al., 1989). Since phosphorylation occurs because of physiological changes communicated by hormonal or neuronal signals, the three tissue-specific isozymes respond differently to this mode of regulation (Agius 2015). In lGP, phosphorylation is the main regulatory mechanism because of the function of lGP in maintaining plasma glucose levels (Wolf et al., 1970). In mGP, phosphorylation helps activate the enzyme with the onset of exercise, but modulator binding (e.g., AMP, ATP, glucose, glycogen, and caffeine) provides another level of control based on cellular energy levels (Howlett et al., 1998). Lastly, since brain glycogen is an emergency glucose store, bGP is tightly regulated solely by modulator binding to respond to hypoxic stress and support high cognitive processes (Mathieu et al., 2016).

#### 2.5.2 AMP-Binding Site

All three isozymes have a well-conserved AMP-binding site that is formed by a bundle of helices located at the interface between dimer subunits (Figure 6A) (Sprang et al., 1991; Rath et al., 2000a). However, isozyme-specific amino acid substitutions within the site lead to differential binding affinity, thus affecting allosteric control (Hudson et al., 1993; Mathieu et al., 2016). This site is also known as the allosteric site because it contains three subsites (sugar, nucleotide base, and phosphate) that allows for promiscuous effector binding (e.g., AMP, ATP, and G6P). Because of the low level of specificity, the nature of the bound effector determines whether it exerts inhibition or activation of enzyme function (Wang et al., 1970). Since the position of the AMP site allows for effectors to bind between subunits, they are often associated with quaternary structural changes (Sprang et al., 1991).

The function of the AMP site corresponds with intracellular energy demands. As energy needs increase, the intracellular concentration of AMP increases because of ATP hydrolysis. AMP binds in this site to stabilize the active relaxed state of GP (R state) that allows for access to the catalytic site (Barford and Johnson 1989). ATP and G6P can then displace AMP and destabilize quaternary interactions to switch the enzyme conformation back to the less active tense state (T state) (Kasvinsky 1982; Sprang et al., 1987; Gaboriaud-Kolar and Skaltsounis 2013). Importantly, Ser14 phosphorylation complements AMP site binding by inducing conformational changes that bury the AMP site and increase effector binding through additional intermolecular forces (Barford et al., 1991).

#### 2.5.3 Glycogen-Binding Site

The GP oligomeric state is an important facet in mediating GP activity. Homodimeric GP is the active form of the enzyme, but activation by AMP or Ser14 phosphorylation causes a change in the tertiary and quaternary structures of the enzyme to promote pairs of dimers to form tetramers. Tetramerization partially blocks access to the active site, thus decreasing enzyme activity to 12–33% of the fully active dimers (Huang and Graves 1970). Furthermore, formation of the tetrameric interface leads to global structural changes that affect the propagation of other allosteric effects (Barford and Johnson 1992). The glycogen storage site is located ∼30 Å from the catalytic site and is situated at the entrance of the catalytic tunnel (Figure 6A). This site forms a contact for the tetrameric interface, so binding of glycogen discourages further oligomerization and leaves the enzyme as an active homodimer (Figure 6B).

#### 2.5.4 Inhibitory Site and Indole-Binding Site

Two additional effector binding sites have been described, the inhibitory site (also known as the nucleoside site or the purine site) and the indole site. Both sites inhibit glycogen phosphorylase by stabilizing the T state and working synergistically with other GP inhibitors (Kasvinsky et al., 1978a; Martin et al., 1998). The inhibitory site is a hydrophobic binding pocket ∼10 Å from the active site (Figure 6A). Its low specificity allows for binding to a diverse set of ligands, including purines, nucleosides, nucleotides, and other related heterocyclic compounds (Oikonomakos et al., 2000a). Inhibitory site ligands interact with a loop called the 280s loop that forms a gate to block substrate access to the active site. Communication with the AMP-binding site decreases AMP binding to hinder GP activation (Kasvinsky et al., 1978b; Buchbinder and Fletterick 1996; Ekstrom et al., 2002). The indole site was first discovered in human lGP during a screening of antidiabetic agents targeting GP (Rath et al., 2000b). The natural ligand is unknown, but synthetic effector binding forms a bridge between dimeric subunits, thus stabilizing the less active conformation and inhibiting GP. Without ligand binding at the indole site, the cavity is instead solvent-filled, which allows for the necessary rotation of subunits during activation (Ercan-Fang et al., 2005).

### 2.6 Fold Types VI and VII

Homology searches identified many PLP-dependent enzymes belonging to the above five fold types. However, other PLP-dependent enzymes that do not resemble the archetypal enzymes were also discovered and subsequently categorized into new fold types (Percudani and Peracchi 2003). Fold Types VI and VII are sparsely populated but contain examples of allostery. Lysine fermentation in anaerobic bacteria uses two analogous PLP-dependent enzymes, lysine 5,6-aminomutase (5,6-LAM) and lysine 2,3-aminomutase (2,3-LAM), to catalyze non-classical, free radical reactions (Ballinger et al., 1992; Chang and Frey 2000). Although both aminomutases have similar reaction mechanisms and analogous intermediates, their structures differ enough to organize into Fold Types VI and VII.

#### 2.6.1 Fold Type VI: Lysine 5,6-Aminomutase (5,6-LAM)

**FIGURE 7:**
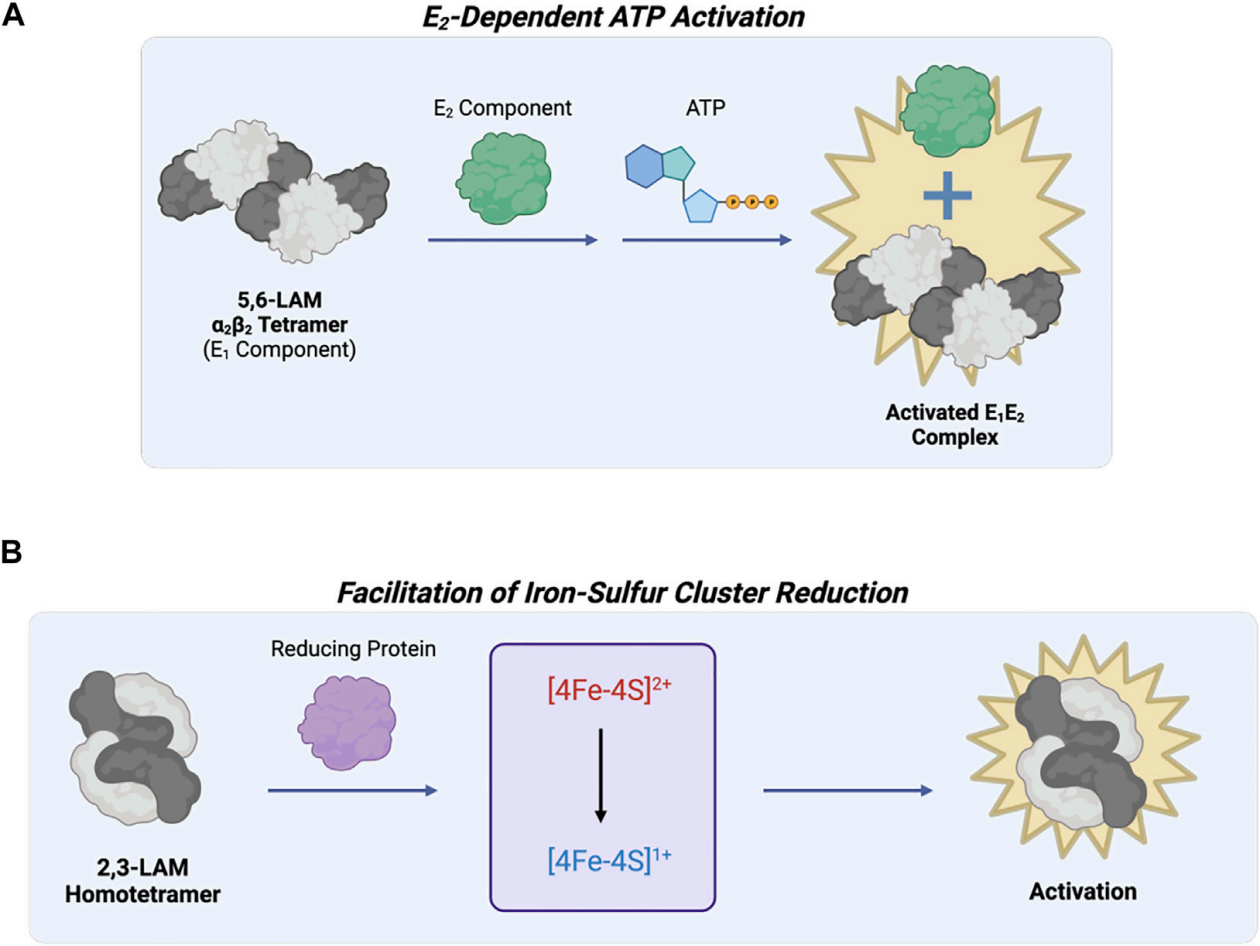
Lysine 5,6-aminomutase of Fold Type VI and Lysine 2,3-aminomutase of Fold Type VII are involved in the same metabolic pathway but are differentially regulated. **(A)** 5,6-LAM (Fold Type VI) is active as an α_2_β_2_ tetramer (known as the E_1_ component), but reactivation and ATP regulation are possible through interaction with another protein referred to as the E_2_ component. **(B)** 2,3-LAM (Fold Type VII) uses three cofactors, PLP, AdoMet, and [4Fe-4S]^+^, for catalysis. AdoMet and [4Fe-4S]^+^ work together for adenosyl radical formation, but this step is limited by 4Fe-4S cluster formation and reduction to the +1 state. Interaction between 2,3-LAM and a reducing protein (e.g., flavodoxin NADP+ reductase, flavodoxin, or ferredoxin) activates 2,3-LAM.

Lysine 5,6-aminomutase (5,6-LAM) catalyzes the reversible 1,2 rearrangement of the terminal amino group of DL-lysine and L-β-lysine. Unlike other PLP-dependent enzymes, 5,6-LAM requires radical propagation from another cofactor, adenosylcobalamin (AdoCbl; vitamin B12), to the external aldimine for its reaction mechanism to occur. This protein forms an α_2_β_2_ tetramer (a dimer of αβ units) (Berkovitch et al., 2004), and the complete holoenzyme is composed of the tetramer in addition to an auxiliary activating protein (Figure 7A). The 5,6-LAM tetramer is known as the active core enzyme E_1_ that uses the coenzymes PLP and AdoCbl. The other component, a sulfhydryl protein E_2_, is responsible for the reactivation and ATP-dependent allosteric regulation of E_1_ (Baker et al., 1973; Chang and Frey 2000). The function of E_2_ may be tied to the exchange of free AdoCbl with bound cobalamins to reactivate the holoenzyme (Toraya and Mori 1999). The PLP- and AdoCbl-dependent D-ornithine 4,5-aminomutase (4,5-OAM) is structurally similar and has a similar reaction mechanism to 5,6-LAM (Barker 1981). 4,5-OAM is an α_2_β_2_ heterotetramer made of the catalytic β subunit and the α subunit necessary for folding (Chen et al., 2001; Wolthers et al., 2008). The α subunit can form a complex with the 5,6-LAM heterotetramer to restore ATP regulation and may work together with E_2_ to reactivate E_1_ (Tseng et al., 2007; Wolthers et al., 2008).

#### 2.6.2 Fold Type VII: Lysine 2,3-Aminomutase (2,3-LAM)

Lysine 2,3-aminomutase (2,3-LAM) was the first aminomutase to be discovered (Chirpich et al., 1970). It is a homotetramer (a dimer of dimers) that catalyzes the interconversion of L-α-lysine and L-β-lysine using PLP, AdoMet, and a [4Fe-4S]^+^ cluster as coenzymes (Lepore et al., 2005). Most isomerization reactions require the use of AdoCbl as a coenzyme, but 2,3-LAM uses AdoMet and a [4Fe-4S]^+^ cluster to mediate hydrogen transfer by radical propagation. Since AdoMet is not as easily cleaved as AdoCbl, the iron-sulfur cluster is needed as an electron source to convert AdoMet into methionine and an Ado radical (Baraniak et al., 1989; Ballinger et al., 1992). Because of the essential nature of the iron-sulfur cluster, 2,3-LAM is catalytically limited by cluster formation and reduction to the +1 state. Previous work showed reducing proteins (e.g., flavodoxin NADP^+^ reductase, flavodoxin, and ferredoxin) can help activate 2,3-LAM by facilitating cluster reduction (Figure 7B) (Brazeau et al., 2006).

## 3 Discussion

Pyridoxal 5′-phosphate is a coenzyme involved in a number of essential cellular processes within nearly all organisms. The diversity of reactions catalyzed by PLP-dependent enzymes is not fully realized solely by examination of their 3-D structures and active site architectures. Rather, this review provides an overview of the diverse mechanisms of protein allostery utilized by members of PLP-dependent enzyme families. It is through allostery that PLP-dependent activities are tuned to meet a particular cellular need. Although there are some reoccurring themes, such as oxidation of a CXXC motif or regulation via an N-terminal or C-terminal extension, there are also many divergent mechanisms. These mechanisms include binding of diverse protein or small molecule effectors at distal sites which induce conformational changes that alter oligomerization or active site accessibility and architecture. Here, we present allosteric mechanisms from various members of each fold type. However, as structural and biochemical investigations continue, it is likely that more allosteric routes will be discovered.

A common approach in drug development is the design of competitive inhibitors that directly disrupt catalysis by active site interference. This concept proves difficult with PLP-dependent enzymes because of the common reaction mechanisms within fold types. How can we target a particular enzyme without affecting a litany of other biological processes? Nature surmounts this problem by the evolution of allosteric regulation, where structural changes distal to the active site induce conformational changes to fine-tune catalysis. Allostery does not center around a certain mechanism but rather encompasses a multitude of means. By exploiting the natural phenomenon of allostery, the issue of active site similarity can be circumvented, and PLP-dependent enzymes that have been previously structurally characterized can be reexamined for novel therapeutic development. Not only will this expand drug discovery opportunities, but it will also aid in exploring novel structure-based means of allosteric regulation.
