# Supplementary material for: Structural Basis for Allostery in PLP-dependent Enzymes
Source: Front Mol Biosci. 2022 Apr 25;9:884281. doi: 10.3389/fmolb.2022.884281 (PMC9081730; doi:10.3389/fmolb.2022.884281)
Supplement: Supplementary file 1 [file Table1.docx]

**Supplemental Table 1. Examples of PLP-dependent enzymes and known allosteric mechanisms**

| **Enzyme** | **Pathway** | **Oligomeric State** | **Allosteric Mechanism** | **References** |
| --- | --- | --- | --- | --- |
| **Fold Type I** |  |  |  |  |
| Aminolevulinic acid synthase (ALAS) | Tetrapyrrole biosynthesis (animals, yeast, α-proteobacteria) | Homodimer | In the human erythroid-specific isoform (ALAS2), access to the active site is occluded by steric hindrance from the C-terminal extension, which forms a salt-bridge network with the active site loop. | (Bailey et al. 2020) |
| Arginine decarboxylase (AdiA) | Bacterial and viral acid stress response system | Homodimer or Decamer (pentamer of homodimers) | Arginine decarboxylase is specifically known as acid-induced arginine decarboxylase (AdiA). At low pH conditions, the surface charges of homodimeric units are neutralized, and AdiA forms catalytically active decamers. | (Andrell et al. 2009) |
| Dialkylglycine decarboxylase (DGD) | α,α-dialkylamino acid degradation | Tetramer (dimer of homodimers) | DGD has two metal ion binding sites, one of which helps regulate catalysis. Site 1 near the active site can interact with monovalent cations with ionic radii of 1.3 to 1.5 Å to activate DGD by promoting cofactor binding and organization of the active site. Binding of smaller monovalent cations to Site 1 causes inhibition. | (Toney et al. 1993, Hohenester et al. 1994, Toney et al. 1995) |
| DOPA decarboxylase (DDC; AKA Aromatic amino acid decarboxylase, AADC) | Epinephrine biosynthesis | Homodimer | DDC has a flexible loop containing a conserved tyrosine residue. This loop regulates catalysis in *trans* by extending toward and occluding the active site of the neighboring monomer upon substrate binding. This closed conformation stabilizes reaction intermediates and allows tyrosine to play a part in the reaction mechanism. In plant AADCs, replacement of the conserved tyrosine with phenylalanine can convert enzyme activity from decarboxylation only to decarboxylation-deamination. | (Bertoldi et al. 2002, Giardina et al. 2011, Bertoldi 2014) |
| Glutamic acid decarboxylase (GAD) | GABA biosynthesis | Homodimer | The 65 kDa isoform of GAD (GAD65) can auto-inactivate through flexibility of a catalytic loop. The GAD65 loop has a similar mechanism as the DDC loop by acting in *trans* from one monomer to its neighbor to contribute a tyrosine residue important in the reaction mechanism. Phosphorylation also differentially regulates GAD activity in the brain by activating GAD65 but inhibiting GAD67. | (Fenalti et al. 2007, Langendorf et al. 2013, Chou et al. 2017) |
| Glutamate-1-semialdehyde aminomutase (GSAM) | Tetrapyrrole biosynthesis (plants, green algae, most bacteria) | Homodimer | GSAM has an active site “gating loop” that acts within the same subunit to regulate access for substrate binding in a cofactor-dependent manner. Initially, GSAM is bound to PMP, and the gating loop is in the open conformation to allow product release and substrate access. As the reaction progresses, PMP converts to PLP, and the loop adopts a closed conformation to occlude the active site. Since substrates bind successively, GSAM can simultaneously bind PMP and PLP, causing negative cooperativity. GSAM has also been reported to form a complex with glutamyl-tRNA reductase (HemA) to facilitate substrate channeling from HemA to GSAM. | (Hennig et al. 1997, Stetefeld et al. 2006, Nardella et al. 2019) |
| Phosphoserine aminotransferase (PSAT) | Serine biosynthesis | Homodimer | PSAT has a “gating loop” that acts within the same subunit to occlude access to the active site. The loop is first in its open conformation where it can guide substrates toward the catalytic site and orients them for catalysis. Upon substrate binding to the active site, the loop adopts a closed conformation to stabilize the substrate. | (Sekula et al. 2018) |
| Serine hydroxymethyl  transferase (SHMT) | Deoxythymidylate (dTMP) biosynthesis | Tetramers (dimer of homodimers) or Homodimers in eukaryotes and bacteria | Mammalian SHMT isoforms function as tetramers that are formed as dimers of obligate dimers. The tetrameric interface is only available when the obligate dimer units adopt a closed conformation. PLP binding in mitochondrial SHMT (SHMT2) causes folding of unstructured regions near the active site that drives the dimer to favor the closed conformation, thus shifting toward tetramerization to lock in the catalytically active closed dimer. A conserved flap motif of one monomer links to the active site of the opposite monomer in an obligate dimer. This motif is therefore integral to stabilizing the tetrameric state through facilitating PLP and substrate binding. | (Giardina et al. 2015, Ubonprasert et al. 2019) |
| Serine palmitoyltransferase (SPT) | Sphingolipid biosynthesis | Heterodimer (SPTLC1 and SPTLC2) | The SPT heterodimer associates with additional regulatory units. Small subunits of serine palmitoyltransferase (ssSPTs) activate SPT nearly 100-fold and dictate substrate preference for acyl-CoA. On the other hand, orosomucoid-like (ORM) proteins negatively regulate SPT. Recent cryoEM structures of the human SPTLC1-SPTLC2-ssSPTa-ORMDL3 complex have shown that ssSPTa shapes the substrate-binding tunnel of SPT, whereas ORMDL3 blocks the tunnel and competes with substrate binding. | (Siow and Wattenberg 2012, Harmon et al. 2013, Wang et al. 2021) |
| Tryptophanase (Trpase) | L-tryptophan metabolism; Nitrogen metabolism | Tetramer (dimer of homodimers) | Trpase binds Rcd, which is a short, untranslated RNA involved in cell division. Through this interaction, Trpase tryptophan affinity is increased, leading to tryptophan degradation and subsequent indole production that delays cell division. Trpase also contains a cation-binding site whereby the identity of the bound cation modulates activity. Binding of smaller ions (Li^+^ and Na^+^) leads to inhibition. | (Snell 1975, Isupov et al. 1998, Chant and Summers 2007, Tsesin et al. 2007) |
| **Fold Type II** |  |  |  |  |
| Cystathionine β-synthase (CBS) | Transsulfuration; H_2_S biosynthesis | Tetramer (dimer of homodimers) | Heme acts as a chaperone for CBS folding. Addition of heme to CBS growth media promotes the formation of active CBS tetramers.  The regulatory domain of one CBS protomer can block the active site of another protomer. AdoMet binding to the regulatory domain will open access to the active site by moving the regulatory domain.  CBS is regulated by oxidative stress by two proposed methods: gaseous signaling molecule binding to ferrous heme or disulfide formation of the CXXC motif. | (Janosik et al. 2001, Taoka et al. 2002, Majtan et al. 2008, Ereno-Orbea et al. 2013, Niu et al. 2018) |
| O-acetylserine sulfhydrylase (OASS) | L-cysteine biosynthesis [sulfur metabolism] | Homodimer | Anion binding (i.e., sulfide) to the OASS allosteric site at the dimeric interface causes a conformational change that moves the substrate binding loop to prevent formation of the external aldimine, causing partial competitive inhibition. The C-terminal tail of serine acetyltransferase (SAT) also inhibits OASS activity by blocking the active site and stabilizing the closed conformation with decreased substrate binding. OASS displays a “competitive-allosteric” mechanism by which residue M120, located ~20Å away from the active site, selectively recruits substrate, enhancing its affinity and in turn leading to dissociation of SAT. | (Burkhard et al. 2000, Tai et al. 2001, Campanini et al. 2005, Singh et al. 2017, Kaushik et al. 2021) |
| Serine racemase (SR) | D-serine biosynthesis | Homodimer | SR is activated by divalent cations (e.g., Mg^2+^, Mn^2+^, Ca^2+^) and nucleotides (e.g., ATP, ADP, and GTP). ATP binds at a site located on the dimer interface. Through an extensive hydrogen bonding network, ATP interacts with the active site to orient catalytic residues, thus activating SR. Mg^2+^ can then interact with the ATP phosphate groups and further activate SR by promoting formation of a more active tetramer. ATP activation is predominately due to stabilizing the closed form of the external aldimine, which can allosterically modulate malonate inhibitor binding at the active site ~20Å away from the ATP binding site. A Ca^2+^-ATP complex can also form but is less efficient at activation. A separate metal binding site accommodates divalent cations to stabilize the protein and shift oligomerization toward a tetramer. SR is also known to interact with proteins that associate with AMPA and NMDA receptors, such as GRIP and PICK1, which leads to modulation of SR activity. | (Kim et al. 2005, Fujii et al. 2006, Marchetti et al. 2015, Bruno et al. 2017, Canosa et al. 2018, Raboni et al. 2018) |
| Threonine deaminase (TDA) | Branched-chain amino acid (isoleucine) biosynthesis | Tetramer (dimer of homodimers) | TDA is one of the first proteins found to exhibit end- product feedback inhibition. TDA exists in equilibrium between a tense (T) state and a relaxed (R) state. Addition of its substrate, threonine, shifts TDA to the R-state for catalysis. However, isoleucine, the end-product, acts as an inhibitor by stabilizing the T-state and forming a second ligand binding site. Ile may bind at the second site and cause further inhibition by changing the conformation of the active sites. Valine, a product from a parallel pathway, counters the inhibitory effect by binding to the second site and reducing affinity of Ile at the first site. Both effectors interact with the C-terminal regulatory domain of TDA, which is connected to the N-terminal catalytic domain by a thin neck-like region. | (Umbarger and Brown 1958, Changeux 1963, Gallagher et al. 1998, Shulman et al. 2008, Chen et al. 2013) |
| Threonine synthase (TS) | Threonine biosynthesis | Homodimer | Plant TS is activated by AdoMet, which is the end-product of a parallel pathway using the same substrate. AdoMet binding to an allosteric site increases the rate of catalysis and substrate binding through reorganization of active site loops. The two AdoMet binding sites within a dimer are slightly different, leading to differences in site stability and dissimilar domain movements for each monomer. However, substrate binding in one monomer triggers successive AdoMet binding in the second monomer. | (Curien et al. 1998, Mas-Droux et al. 2006) |
| Tryptophan synthase (TrpS) | L-tryptophan biosynthesis | α_2_β_2_ Tetramer (dimer of αβ heterodimers) | The TrpS reaction proceeds first with the α subunit (TrpA), where indole is produced and funneled into the β subunit (TrpB) for the PLP-dependent β-replacement reaction. The active sites of the different subunits are distant to one another, so they do not affect each other directly. Instead, the formation of the α_2_β_2_ tetramer helps to stabilize TrpB and channel the indole substrate through a tunnel. The primary interacting residues between the subunits are the α loop 2 and β COMM domain. Binding of ligands to TrpA triggers a closed conformation that extends toward TrpB to cause the rigid β COMM domain to block escape of substrate and promote catalysis. This interaction causes the isolated components to be inefficient without one another. Inactive TrpA remains an allosteric activator of TrpB by increasing TrpB activity four-fold.  TrpS is also activated by monovalent cations that bind to TrpB near the active site. These cations cause local changes to the cation site (depending on the cation), which propagate to the interaction site between subunits and alter the substrate channeling tunnel. Recent studies used computational methods to identify TrpS allosteric networks and facilitate development of allosteric inhibitors. | (Hyde et al. 1988, Rhee et al. 1996, Schneider et al. 1998, Miles et al. 1999, Dunn 2012, Miles 2013, Buller et al. 2015, Maria-Solano et al. 2019, D'Amico et al. 2021, Ghosh et al. 2021, Michalska et al. 2021, O'Rourke et al. 2021, Phillips and Harris 2021, Bosken et al. 2022) |
| **Fold Type III** |  |  |  |  |
| Alanine racemase (AR) | D-alanine biosynthesis [bacterial cell wall biosynthesis] | Homodimer | Recent data suggests that the AR homodimer contains two surface pockets that can interact with small molecules. N-terminal lid regions close over the pockets to sequester modulators in a closed conformation. | (Jyothikumar et al. 2018) |
| Ornithine decarboxylase (ODC) | Polyamine biosynthesis | Homodimer | Small inhibitory proteins called antizymes act as regulators of polyamine biosynthesis. The most prominent, antizyme 1 (AZ1), binds to monomers of eukaryotic ODC to block ODC dimerization and promote proteasomal degradation of ODC in a ubiquitin-independent manner. | (Mangold 2005, Wu et al. 2015) |
| **Fold Type IV** |  |  |  |  |
| Branched-chain amino acid aminotransferase (BCAT) | Branched-chain amino acid metabolism | Homodimer | Contains a CXXC oxidoreductase motif that may be used for oxidative stress sensing and enzymatic inhibition in the human mitochondrial isoform. C315 acts as the redox sensor, and C318 is the “resolving cysteine” that can form a reversible disulfide bond under oxidizing conditions. | (Conway et al. 2004, Yennawar et al. 2006) |
| **Fold Type V** |  |  |  |  |
| Glycogen phosphorylase (GP) | Glycogenolysis | Homodimer | Each isoform of GP can be differentially regulated based on the various mechanisms. Phosphorylation at Ser14 of the GP homodimer orders the N-terminal 14 amino acids (the phosphorylation peptide) and causes a rotation of subunits, which activates the GP homodimer. Ligand binding at the AMP site can also activate (e.g., AMP) or inhibit (e.g., ATP) GP depending on the nature of the ligand. Since the AMP site is located at the dimer interface, quaternary interactions are affected by ligand binding. Both phosphorylation and AMP binding trigger GP tetramerization, which inhibits GP by blocking the active sites. However, tetramerization can be blocked by glycogen binding at the tetrameric interface. GP has two other ligand binding sites, the indole site located at the dimer interface and the inhibitory site near the active site. Ligand binding at the indole site destabilizes dimerization. Ligands in the inhibitory site interact with the 280’s loop that forms a gate for the active site, thus limiting substrate access. | (Barford and Johnson 1989, Barford et al. 1991, Oikonomakos et al. 2000, Rath et al. 2000, Gaboriaud-Kolar and Skaltsounis 2013) |
| **Fold Type VI** |  |  |  |  |
| Lysine 5,6-aminomutase (5,6-LAM) | Lysine degradation | Tetramer (dimer of αβ dimers) | The 5,6-LAM complex is made of two components, the core 5,6-LAM α_2_β_2_ tetramer (E_1_) and an activating component (E_2_). E_2_ binds ATP, which is needed for free AdoCbl exchange with bound cobalamins. This exchange reactivates the core tetramer. | (Toraya and Mori 1999, Chang and Frey 2000) |
| **Fold Type VII** |  |  |  |  |
| Lysine 2,3-aminomutase (2,3-LAM) | Lysine degradation | Tetramer (dimer of homodimers) | 2,3-LAM uses AdoMet as a cofactor, which needs a [4Fe-4S]^+^ cluster to cleave AdoMet and produce a catalytic 5′-deoxyadenosyl radical. [4Fe-4S]^+^ cluster assembly and reduction from the 2+ state to 1+ are limiting factors of 2,3-LAM catalysis. Reducing proteins (e.g., FPR, FLD, and FD) can facilitate cluster reduction and thus activate 2,3-LAM. | (Brazeau et al. 2006) |
